# The human-snail transmission environment shapes long term schistosomiasis control outcomes: Implications for improving the accuracy of predictive modeling

**DOI:** 10.1371/journal.pntd.0006514

**Published:** 2018-05-21

**Authors:** David Gurarie, Nathan C. Lo, Martial L. Ndeffo-Mbah, David P. Durham, Charles H. King

**Affiliations:** 1 Department of Mathematics, Applied Mathematics and Statistics, Case Western Reserve University, Cleveland, Ohio, United States of America; 2 Center for Global Health and Diseases, School of Medicine, Case Western Reserve University, Cleveland, Ohio, United States of America; 3 Schistosomiasis Consortium for Operational Research and Evaluation, University of Georgia, Athens, Georgia, United States of America; 4 Division of Epidemiology, Stanford University School of Medicine, Stanford, California, United States of America; 5 Yale School of Public Health, Yale University, New Haven, Connecticut, United States of America; Imperial College London, Faculty of Medicine, School of Public Health, UNITED KINGDOM

## Abstract

**Introduction:**

Schistosomiasis is a chronic parasitic trematode disease that affects over 240 million people worldwide. The *Schistosoma* lifecycle is complex, involving transmission via specific intermediate-host freshwater snails. Predictive mathematical models of *Schistosoma* transmission have often chosen to simplify or ignore the details of environmental human-snail interaction in their analyses. Schistosome transmission models now aim to provide better precision for policy planning of elimination of transmission. This heightens the importance of including the environmental complexity of vector-pathogen interaction in order to make more accurate projections.

**Methodology and principal findings:**

We propose a nonlinear snail force of infection (FOI) that takes into account an intermediate larval stage (miracidium) and snail biology. We focused, in particular, on the effects of snail force of infection (FOI) on the impact of mass drug administration (MDA) in human communities. The proposed (modified) model was compared to a conventional model in terms of their predictions. A longitudinal dataset generated in Kenya field studies was used for model calibration and validation. For each sample community, we calibrated modified and conventional model systems, then used them to model outcomes for a range of MDA regimens. In most cases, the modified model predicted more vigorous post-MDA rebound, with faster relapse to baseline levels of infection. The effect was pronounced in higher risk communities. When compared to observed data, only the modified system was able to successfully predict persistent rebound of *Schistosoma* infection.

**Conclusion and significance:**

The observed impact of varying location-specific snail inputs sheds light on the diverse MDA response patterns noted in operational research on schistosomiasis control, such as the recent SCORE project. Efficiency of human-to-snail transmission is likely to be much higher than predicted by standard models, which, in practice, will make local elimination by implementation of MDA alone highly unlikely, even over a multi-decade period.

## Author summary

Infection with blood fluke *Schistosoma* parasites is a major cause of disease burden around the world. Control of schistosomiasis, which is transmitted through intermediate host freshwater snails, is a priority for national and global health programs working in at-risk regions of Africa, the Mideast, Asia, the Philippines, and South America. Program planning often relies on mathematical models to project the impact of different schedules of mass drug administration (MDA) of the anti-schistosomal drug, praziquantel, in these areas. In practice, though, recent projections of standard models have failed to capture the variability of MDA program impact on community levels of infection, especially in high-risk zones. In the present study, we developed a modification of the conventional modeling approach that takes more detailed account of human-to-snail transmission. Inclusion of a revised, nonlinear form for the model’s snail infection function had profound effects on long term predictions of the impact of MDA programs for *Schistosoma* control. In specific, our proposed snail parameters helped to explain the persistent rebound of *Schistosoma* prevalence in certain high risk communities. The efficiency of human-to-snail transmission is likely to be much higher than predicted in standard models, which makes local elimination by implementation of MDA, alone, highly unlikely.

## Introduction

Schistosomiasis is a neglected tropical disease (NTD) having an estimated global prevalence of 240 million infected persons, many of whom experience significant morbidity within the infected communities of Africa, the Mideast, South America, Asia, and the Philippines [1]. For global control of the disease schistosomiasis, the World Health Organization (WHO) recommends delivery of the anti-helminthic drug, praziquantel, via mass drug administration (MDA), with attempts at local elimination, where possible [1, 2]. Unlike the very effective MDA experience obtained for other helminthic NTDs such as onchocerciasis and lymphatic filariasis [3, 4], there remain significant concerns about the feasibility of schistosomiasis elimination using MDA alone [5]. This is in part due to that fact that MDA has been unable to interrupt schistosomiasis transmission in many endemic areas, even after a decade or more of repeated MDA [6, 7]. This failure to interrupt transmission has often been marked by a significant rebound of infection prevalence following termination of MDA [8–10], or of concurrent mollusciciding interventions [11]. The highly uneven landscape distribution of suitable intermediate host snail habitat, combined with weather- and climate-related seasonal differences in snail abundance, mean that there is often a quite varied patchwork of transmission zones within any given region slated for parasite control [12–16].

Understanding the mechanisms that drive infection rebound is crucial for the development and implementation of more efficient control strategies [1]. Conventional predictive models of transmission suggest that where rebound is slow, there can be progressive reduction of parasite burden after each MDA cycle, so we may expect to bring *Schistosoma* burden under control and achieve elimination of transmission [17]. On the other hand, rapid rebound of parasite burden following treatment serves to impede long term progress towards elimination goals, and necessitates additional MDA effort and/or introduction of complementary environmental control measures to achieve parasite elimination [5, 18, 19].

In developing transmission models, important but often overlooked determinants of schistosome transmission are the ecology and population biology of the intermediate snail host and accurate assessment of the human-to-snail force of infection (FOI). As part of the transmission cycle, *Schistosoma* must infect very specific intermediate host snail species, then undergo a process of extensive asexual multiplication within the snail’s body in order to create the free-swimming cercariae that will infect the next round of human hosts [20, 21]. For *Schistosoma* parasites of humans, local presence of freshwater snail species of genera *Bulinus*, *Biomphalaria*, *Oncomelania*, or *Neotricula*, is essential to the transmission of *Schistosoma haematobium*, *S*. *mansoni*, *S*. *japonicum*, and *S*. *mekongi*, respectively [22]. Because snail infection is an obligate stage for parasite transmission, ecological factors that favor the presence and abundance of these ‘vector’ snails also foster local risk for these *Schistosoma* spp. infections and for their related human disease states, either urogenital or intestinal schistosomiasis [15, 20, 23].

**Fig 1:**
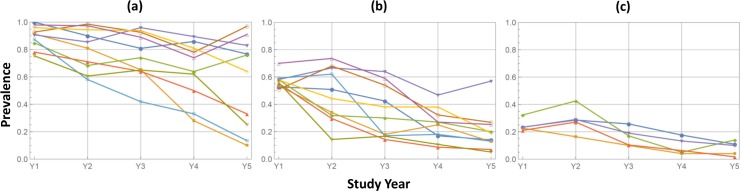
Differential response to MDA in multi-year control study of *S*. *mansoni* in western Kenya (SCORE project [27, 29]). School age children (SAC) ages 9–12 were screened annually in a group of 25 study villages given annual community-wide praziquantel treatment. SAC treatment coverage levels were >80% and were comparable for all villages, but the observed infection prevalence responses were highly uneven. In partitioning these 25 communities into 3 categories based on their baseline pre-intervention (Y1) SAC prevalence levels, (a) High (> 75%); (b) Medium (50% -74%); (c) Low (<50%), the uneven response was particularly noticeable in the high and medium prevalence categories. This phenomenon was not predicted by standard dynamic models [10, 17].

Conventional transmission models assume the snails’ FOI is a linear function of human infectivity (see, *e*.*g*. [24–26]). Under this assumption, any drop in human infectivity (e.g. via MDA-related reduction in local egg excretion), will *proportionately* reduce the rate of local snail infections, which in turn will slow reinfection of human hosts. However, the empiric field data from recent large-scale, cluster- randomized operational research trials of anti-schistosomal MDA [27] have demonstrated a broad range of community-level parasitological responses, ranging from highly effective reductions in prevalence and intensity at some locations, to the existence of highly resistant “hotspots” (Fig 1), where infection levels persist at or near baseline levels despite effective implementation of MDA [28, 29]. While the current simplified deterministic models mimic the average effects of MDA across all communities, the failure to account for broad village-by-village variability is a challenge to the general utility of transmission model-based predictions.

Prior modeling studies of other micro-and macro-parasite systems have established that the form assumed for the transmission coefficient (beta) can have a significant impact on the projected outcomes for disease ecology models [30–32]. As a simplifying initiative, most modelling approaches typically assume ‘density-dependent’ kinetics, in which two well-mixed populations provide a constant per capita rate of exposure [33]. However, some models have elected to employ ‘frequency-dependent’ kinetics, which can exhibit saturation of transmission at higher host densities or in the presence of non-random mixing [30, 34, 35]. In such models, landscape patchiness, associative movement networks, time-dependence, and heterogeneity in host susceptibility can explain the failure of standard ‘mass action’ transmission coefficients to accurately capture the trajectory of disease transmission in real-world settings [30, 33, 36–38]. These transmission features are common among vector-borne macroparasites such as the *Schistosoma* species studied here. Where such features exist, it is apparent that fine-scale transmission events in linked territories can serve to drive larger meta-population patterns of infection prevalence [30].

To explain observed heterogeneities in *Schistosoma* transmission, we undertook a closer examination of the intermediate snail host and its infection by humans. *Schistosoma* transmission and parasite development have multiple time scales, ranging from “fast” larval dynamics (hours, days), to “slow” (month, years) host-parasite-snail dynamics. In the current study, we focused on these slower dynamics, so larval stages did not enter our model formulation explicitly. However, we saw the need to have an accurate account of their effect on human and snail infection. Conventional modeling approaches assume each FOI to be proportional to its source infectivity and population size [33]. We reexamined the conventional model assumptions, and derived a newer formulation of human-to-snail FOI that combines human host infectivity, demographics and snail population inputs. Among other salient features of the proposed FOI is its nonlinear dependence on human egg output. This functional form could be linked to the magnitude of the post-MDA prevalence rebound and to the consequent success or failure of long-term control.

We explored the effect of modifying snail FOI in simulating MDA responses for typical endemic communities, comparing “nonlinear” vs. “linear” models. The two models produced markedly different outcomes, particularly in high-intensity transmission settings.

## Methods and models

### Human and snail force of infection

The schistosome parasite maintains a complex life cycle, transiting between human and snail hosts, with the transition mediated by two larval stages, the egg-derived miracidium (for human-to snail movement), and the snail-derived cercaria (for snail-to-human movement) [21]. For this study, we applied a previously developed dynamic model that describes this biological process. We denote the corresponding forces of infection *λ* (for snail-to-human), and Λ (for human-to-snail). The former (*λ*) represents the mean rates of worm accumulation by human hosts, the latter (Λ), the mean rate of snail invasion by miracidia.

Each force depends on its host carrier’s infectivity, population abundances, and the frequency and pattern of their contact (human water exposure and water contamination rates). In our setup, human FOI is proportional to infected snail prevalence (0 < *y* < 1), *λ* = *A y*, with transmission coefficient A. Snail FOI is a function of human infectivity, *E* (mean egg release), but its derivation requires careful analysis. Most conventional models employ linear Λ = *B E* with transmission coefficient B [25, 26, 39]. Here, instead, we propose a nonlinear (saturable) form of snail FOI,

$$\Lambda =\Lambda_{0}\left(1-e^{-bE}\right)$$ (1)

The derivation of (1) is outlined in the supporting information S1 File. It employs some natural assumptions on miracidial dynamics from human egg release, its diffusive spread, and the process of snail invasion. We assumed miracidia randomly cluster about snail host, with a Poisson distributed “miracidia/snail” ratio. The resulting saturable (exponential) function (1) is the probability of successful invasion (see S1 File).

To study the effect of nonlinear FOI, we programmed two coupled human-snail model systems, termed M1 (having a linear snail FOI factor), and M2 (having a nonlinear FOI given by Eq (1)). Nonlinear Λ had two coefficients (Λ_0_, *b*) that, through local model calibration, reflected important local environmental, biological, and behavioral inputs. Λ_0_ can be viewed as maximal rate of miracidial invasion in a given environment. It depends on local snail density (which determines “mean travel time” to reach target), and on search strategies employed by miracidia (see [40] for a general discussion of encounter rates). Coefficient *b* is related to the mean miracidia production by human hosts and the probability of snail invasion by miracidia. Additional factors that enter *b* include mean population density (host/snail), and human-snail contact (exposure/contamination) rates.

Different types of human and snail models can be coupled via FOI terms *λ*,Λ. Here we adopted a stratified worm burden (SWB) approach (for the human part), developed in earlier works [41–44], but one can also use a simpler MacDonald-type mean worm burden (MWB) system [24, 45]. The basic differences between models M1 and M2, and their projected control outcomes, are due primarily to the Λ -function, whereas a specific formulation for the human side of the coupled model proved less influential.

Importantly, there can be a convergence between linear and nonlinear FOI systems: Function (1) can be approximated by a linear function

$$\Lambda \left(E\right)\approx \Lambda_{0}bE$$ (2)

at small contagion levels (i.e., *b E* ≪ 1). So our nonlinear Λ (1) can be viewed as an extension of linear form (2) to reflect larger values of human infectivity. Specifically, the M1 and M2 FOIs depart significantly as *E* or *b* grow large; the latter, in particular, embodies higher human-to-snail ratios or higher contact rates. Notably, the two FOI systems can also give markedly different values of transmission coefficients, even when calibrated against the same datasets.

### Snail and human transmission systems

For snail infection modeling, we used a standard simple S-I transmission system (*x–*susceptible (S), *y–*infected (I)) with stationary population density (*x* + *y* = 1). The prevalence variable 0 < *y*(*t*) < 1, solves differential equation

$$\frac{dy}{dt}=\Lambda \left(1-y\right)-\nu y,$$ (3)

with snail FOI, Λ.

For the present analysis, a human SWB model was used, consisting of variables $\vec{h}\left(t\right)=\left\{h_{m}\left(t\right)\right\}$- (worm burden strata) that undergo dynamic changes due to worm accumulation and loss processes. The detailed exposition of SWB approach has been described in detail in previous publications [39, 41, 42], and it is briefly summarized in S1 File.

A conventional MWB setup [39, 41, 42] can also be used if desired. It has a single dynamic variable, MWB *w*(*t*), that obeys differential equation

$$\frac{dw}{dt}=\lambda -\left(\gamma +\mu \right)w$$ (4)

with human FOI (*λ* = *Ay*) depending on snail prevalence (3), and loss term (*γ* + *μ*) which combines worm mortality, *γ*, and host turnover, *μ*. The two models, MWB and SWB, share common input parameters (*λ*,*γ*,*μ*). In fact, MWB Eq (4) follows from the SWB if one takes the first moment (mean) of the {*h*_*m*_}-distribution,

$$w\left(t\right)=\sum_{m>0}^{}mh_{m}\left(t\right).$$

The main difference between the SWB and MWB approaches lies in their assumptions on within-humans worm distribution patterns, {*h*_*m*_}, and the resulting human infectivity *E* (see, *e*.*g*. [41]). The SWB imposes no constraints on variables {*h*_*m*_}, whereas MWB uses *a priori* assumptions to express *E* as a function of *w*(*t*). Typically in the MWB model, {*h*_*m*_}are assumed to follow a negative binomial (NB) with prescribed aggregation constant, k.

In both systems, human infectivity is a product of mean mated worm count (MMC) Φ, and worm fecundity *ρ*, with *E* = *ρ*Φ. The MWB gives MMC as a function of variable *w*, Φ(*w*,*k*), while SWB function $\Phi \left(\vec{h}\right)$ depends on worm burden strata $\vec{h}=\left\{h_{m}\right\}$.

### Coupled human-snail system and model calibration

Two different FOI {*λ*(*y*),Λ(*E*)} couple transmission dynamics between human and snail hosts, and give rise to a coupled SWB-snail model. The setup can be can be extended to demographically-structured populations made of multiple risk/age groups, each carrying specific burden distributions. In our analysis we employed structured host communities made of child (C) and adult (A) age groups, with age-specific FOI and transmission coefficients, *λ*_*C*_ = *A*_*C*_*y*, *λ*_*a*_ = *A*_*a*_*y*.

The combined infectivity of such system depends on MMC Φ_*i*_ of each group, their age-specific worm fecundities *ρ*_*i*_, population fractions (*H*_*c*_ + *H*_*a*_ = 1), and contact (exposure/ contamination) rates *ω*_*i*_. Their combination gives the following dimensionless form

$$E=\rho \left(H_{c}\Phi_{c}+\omega H_{a}\Phi_{a}\right)$$ (5)

Factor ρ_*C*_ is the mean worm fecundity of the child group, while weight *ω* is the product of relative (child-to-adult) fecundity and exposure factors (S1 File). The child age-group worm fecundity is subsumed as a factor in the transmission coefficient, *b*, so doesn’t enter the model explicitly.

Calibration of the coupled systems proceeded in two steps: (i) human egg-count (diagnostic test) data were employed to estimate snail-to-human FOI and worm fecundity (*λ*_*i*_,*ρ*_*i*_) for each human subgroup. The outcome was a best-fit posterior distribution of the model parameter space; (ii) next, the calibrated human parameters were combined with additional environmental/behavioral (snail) data to estimate transmission coefficients *A*_*i*_ (snail-to-human), and either {*B*,*ω*} (for linear FOI), or triplet {Λ_0_,*b*,*ω*} (for nonlinear, Λ; see S1 File, Part B for details).

In our predictions, we used similar snail inputs (baseline prevalence, y*) and the relative adult/child exposure factor, *ω*, in both model systems M1 and M2. However, nonlinear FOI (M2) had an additional parameter, *b*, which encoded the relative human/snail population factor (*H*/*N*). In our sensitivity analysis, we varied *b* to simulate a broad range of environments and explore its effect on MDA outcomes.

### Modeling MDA control

Drug treatment with praziquantel kills a large fraction of adult *Schistosoma* worms, and its clearing efficacy is estimated at 80–95% [5]. In our simulations, we have set this value at 85% (using a surviving worm fraction, *ε* = .15). The key inputs for MDA program simulation consisted of target group sizes (children, adults), their coverage levels (e.g. 0 < *f*_*c*_ < 1, 0 < *f*_*A*_ < 1), and the timing or frequency of MDA delivery (annual, biennial, etc.).

In our numeric simulations, MDA was implemented as an instantaneous event, whereby worm burden of each group is reduced depending on its coverage and drug efficacy, so the dynamical transmission system was reinitialized at time *t*_*d*_ after each control event. For the SWB system, an MDA event results in reshuffling of burden strata, so that each higher-burden stratum shifts to lower-burden strata *h*_*m*_ → *h*_*εm*_ (see [42, 43]). For a corresponding MacDonald-like MWB system, each MDA event with coverage *f*, and efficacy *ε*, would reduce MWB *w*(*t*_*d*_) by a factor *ε f* + (1 − *f*).

### Calibration of the model community

For analysis and MDA simulations we modeled three communities from past Kenyan control-surveillance studies 1983–92 [46], and 2000–2009 [12, 13], having heavy (H), moderate (M) or light (L) infection levels (see Table 1).This dataset was extensively used in our previous SWB work [10], and in more recent papers [41–43, 47]. The latter have employed refined SWB methodology to account for in-host biology (worm mating, aggregation, random egg release), and have introduced more advanced calibration methodologies.

**Table 1:** Baseline infection levels in three surveyed Kenyan communities, classified either as high-, moderate-, or low-intensity transmission zones for *S*. *haematobium* infection.

	Age Group	Transmission Zone		
		High	Moderate	Low
**Prevalence**	Children	69%	32%	24%
	Adults	28%	14%	6.4%
**Mean infection intensity (eggs per 10 mL urine)**	Children	128	93	48
	Adults	21	13	4.5

The modeled high-intensity community (H) was subject to longitudinal study spanning nine years, with two MDA sessions (in 2001 and 2003), and three population-wide surveillance screenings (in 2001, 2003, and 2009). For the purpose of the current comparative modeling analysis, we divided the village population into child (0–20 year old) and adult (20+ years) age groups (Table 2) based on Kenyan demographics. Additional model parameters included in the simulations were worm mortality and snail survival as described in S1 File, Table A1.

**Table 2:** Demographic inputs for the two age strata of the modeled population.

	Children^a^	Adults^a^
**Fraction of population**	49%	51%
**Annual host turnover rate (μ)**	6%	2.5%
**Annual worm loss rate (γ)**	0.2	0.25

^a^ Children = ages zero to 20 years old; adults = over 20 years old

The two study models (M1, M2) were calibrated for each of our high-, moderate- and low-intensity sample communities following [42]. The calibration procedure involved two-steps: (i) individual egg-count test data at baseline (Year 2001) were employed to define a posterior distribution of likely parameter choices (*λ*,*ρ*,*k*) for age-groups C and A. The calibration results (marginal distributions of human parameters and their statistics) are described in S1 File, part B.

The next step used the estimated human parameters (from our first-stage calibration’s posterior distribution) to estimate transmission coefficients. Snail-to-human transmission coefficients *A*_*i*_ (*i* = *C*,*A*) were identical for M1 and M2. The human-to-snail components were different: {*B*,*ω*} for the linear-FOI model M1, and {Λ_0_,*b*,*ω*} for the nonlinear M2. All depended on infected snail prevalence (both prepatent and patent (*i*.*e*., cercaria-shedding)), which was fixed at value y* = 0.3, consistent with PCR-based snail surveillance findings in the Kenyan environment [48]. Patent snail density, which is responsible for transmission, was assumed to be proportional to infected snail prevalence, *y*(t). There were two additional inputs (y*,*ω*) for M1, and 3 additional inputs (y*,*ω*,*b*) for M2. The relative adult/child exposure ratio, *ω*, was set at 1.5, and *b* combined a transmission coefficient (miracidia contagion release the by the child age group) times relative host population abundance (human/snail) (see S1 File, part B). Because these values have been less well studied, in sensitivity analysis we allowed broad range of uncertainties: 0.5 < ω < 5; 0.5 < *b* < 5, for both of these transmission variables.

## Results

### Long term MDA simulations

**Fig 2:**
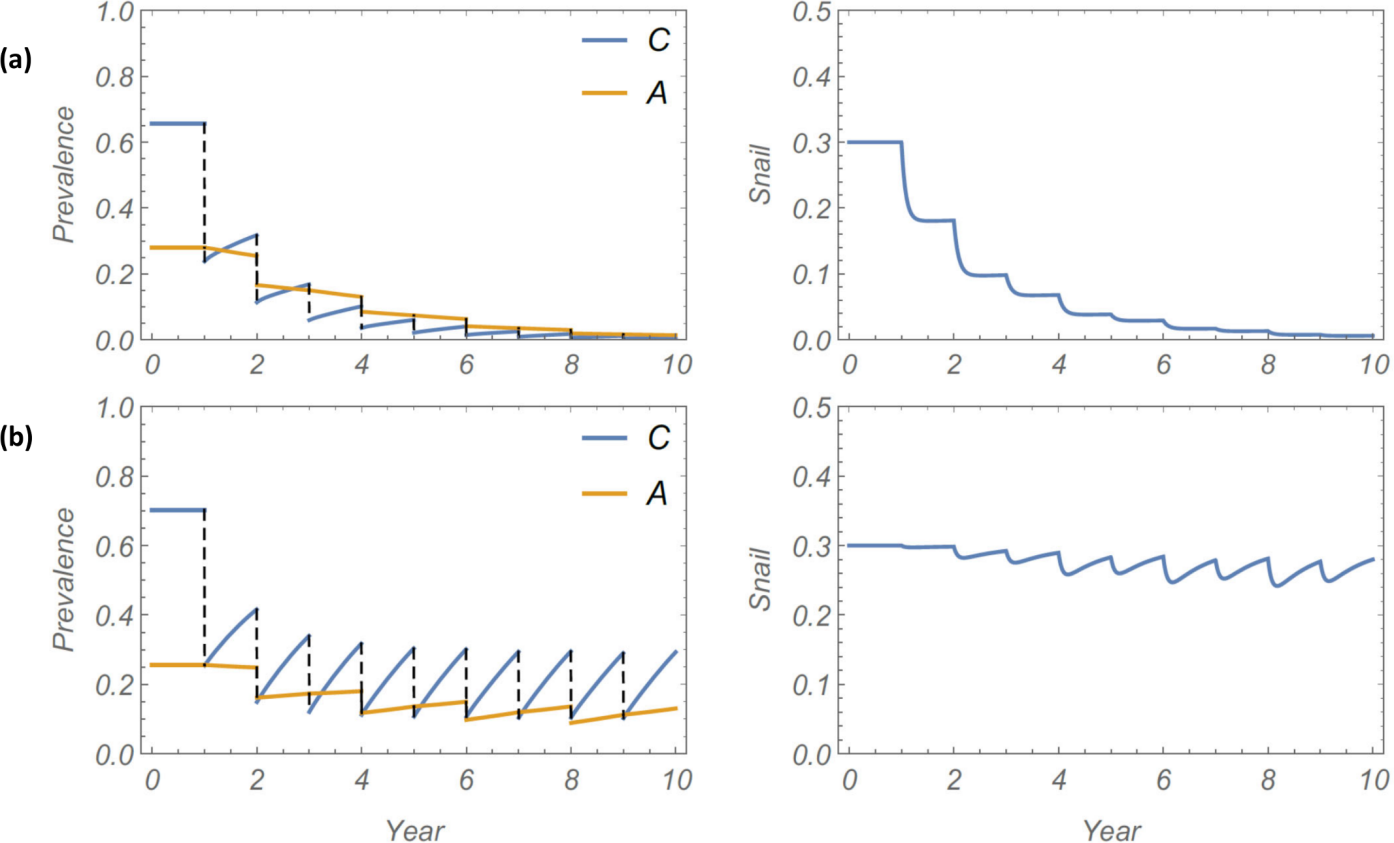
**Projected 10 year MDA history for the calibrated high-risk model community, with either linear (Panel a) or non-linear/saturable FOI (Panel b).** The simulated MDA program attained 75% coverage for children (annually), and 35% coverage of adults (biennially). The left side graphs indicate simulated *S*. *haematobium* infection prevalence over time among children (C) and adults (A); the right side graphs indicate simulated pre-patent + patent infection prevalence among local *Bulinus* snails.

The calibrated model community, using a consistent choice of transmission uncertainties (*y*,*ω*,*b*), was subjected to a series of control experiments to explore the effect of snail FOI assumptions (model M1 vs. M2) and the role of (*y*,*ω*,*b*) on long term MDA outcome patterns in different environmental settings. A typical 10-year history for a high-risk community is shown in Fig 2. The model parameters used in this simulation are listed in Table 3. For this analysis, annual community MDA was used, with an estimated 75% annual coverage for children, and 35% biennial coverage was used for adults.

**Table 3:** Parameters used in long term MDA simulation of a high-risk community.

Variable name	Symbol	Base case value
Snail-to-human FOI for children	λ_C_	2.5
Snail-to-human FOI for adults	λ_A_	0.75
Egg aggregation constant for children	k_C_	0.044
Egg aggregation constant for adults	k_A_	0.032
Worm fecundity within children	ρ_C_	22
Worm fecundity among adults	ρ_A_	11
Relative child:adult exposure ratio	ω	1.5
Basic (child) transmission rate	b	2.2
Infected snail prevalence	y*	0.3

**Fig 3:**
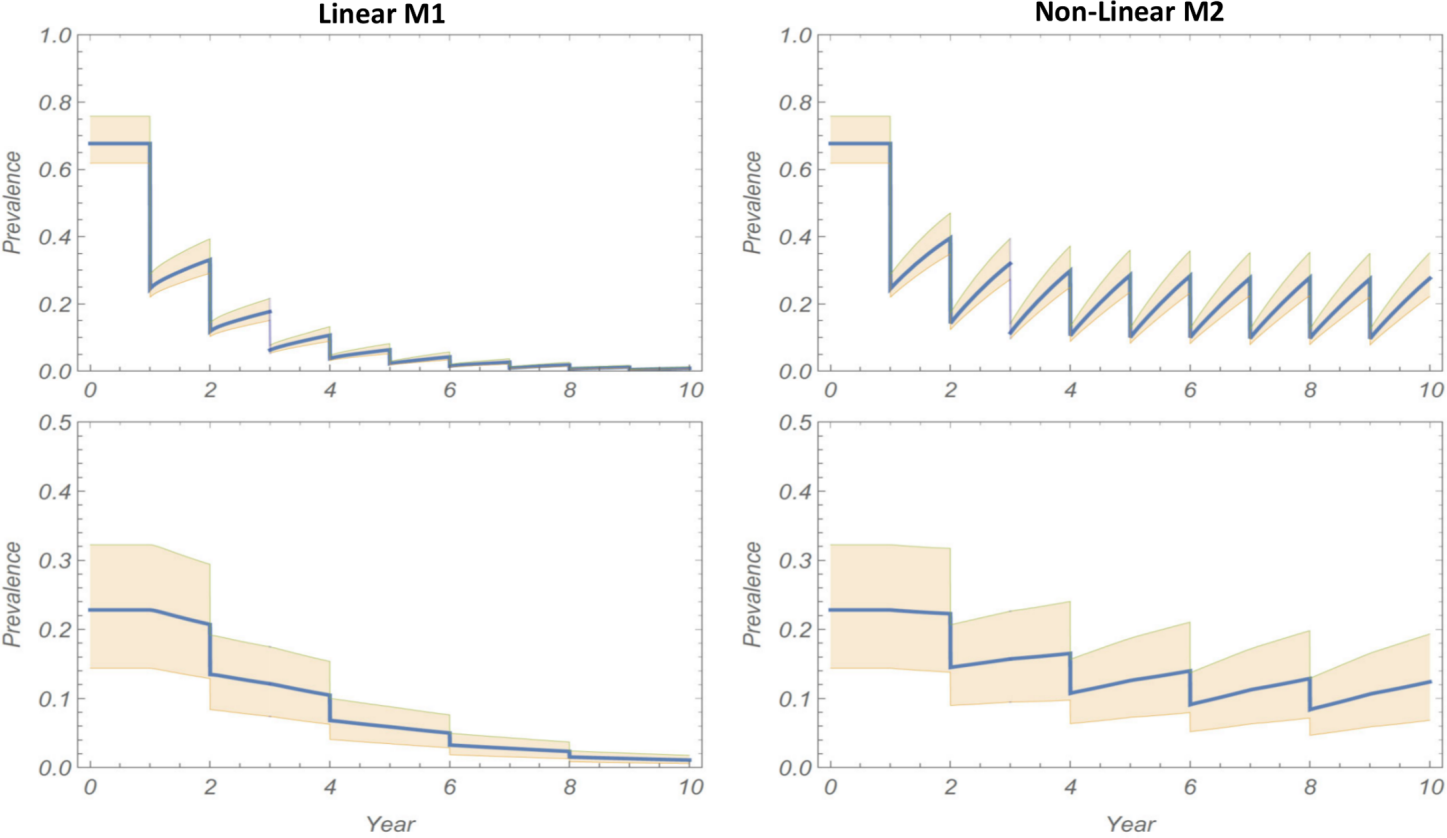
Sensitivity analysis of MDA regimen for the modeled community of Fig 2. Ensemble of 10-year histories over 20 random samples of the M1 and M2 models’ posterior parameter distributions. Left and right columns show respective results (mean history + 95% CI) for the linear M1 model and the nonlinear M2 model types of snail FOI inputs. In each column, the upper and lower graphs display the child and adult prevalence functions, respectively, over time.

The simulation results show large differences between M1 and M2 projections, with the M1 system rapidly approaching elimination, whereas M2 becomes locked in a limit-cycle pattern and does not approach elimination (Fig 2). This qualitative distinction between the models—mainly that M2 model was considerably less likely to achieve MDA-mediated elimination—persisted for a range of parameter choices and MDA coverage. In sensitivity analysis of our prediction by random sampling of model parameters (human and environmental) over a broad range of values with identical M1 and M2 communities subjected to the same MDA regimen, significant differences remained in projected outcomes. History envelopes (Fig 3) show ensemble mean and 95% CI for the multiple simulated 10-year MDA programs. The M1 histories consistently go to elimination, while the M2 histories settle into recurrent limit cycles that fail to achieve elimination.

### Kenyan longitudinal study and model validation

To help validate our approach, we used an observed longitudinal dataset collected over 9-year period for the base case high-risk community, Milalani, in Kwale County, Kenya [46, 49]. The community was screened in (2001, 2003, 2009), with two MDA sessions run in 2001 (community-wide coverage 79%), and in 2003 (community-wide coverage 41%). The results of study are summarized in Table 4.

**Table 4:** Kenyan community study of MDA for *S*. *haematobium* control. Table shows prevalence and intensity of *S*. *haematobium* infection at baseline (2000), year 3 (2003) and year 9 (2009) of the Msambweni project [46, 49].

	Age group	Year		
		2000	2003	2009
**Prevalence**	Children	69%	36%	61%
	Adults	28%	13.5%	20%
**Intensity (eggs per 10 mL urine)**	Children	128	68	133
	Adults	21	21	11

**Fig 4:**
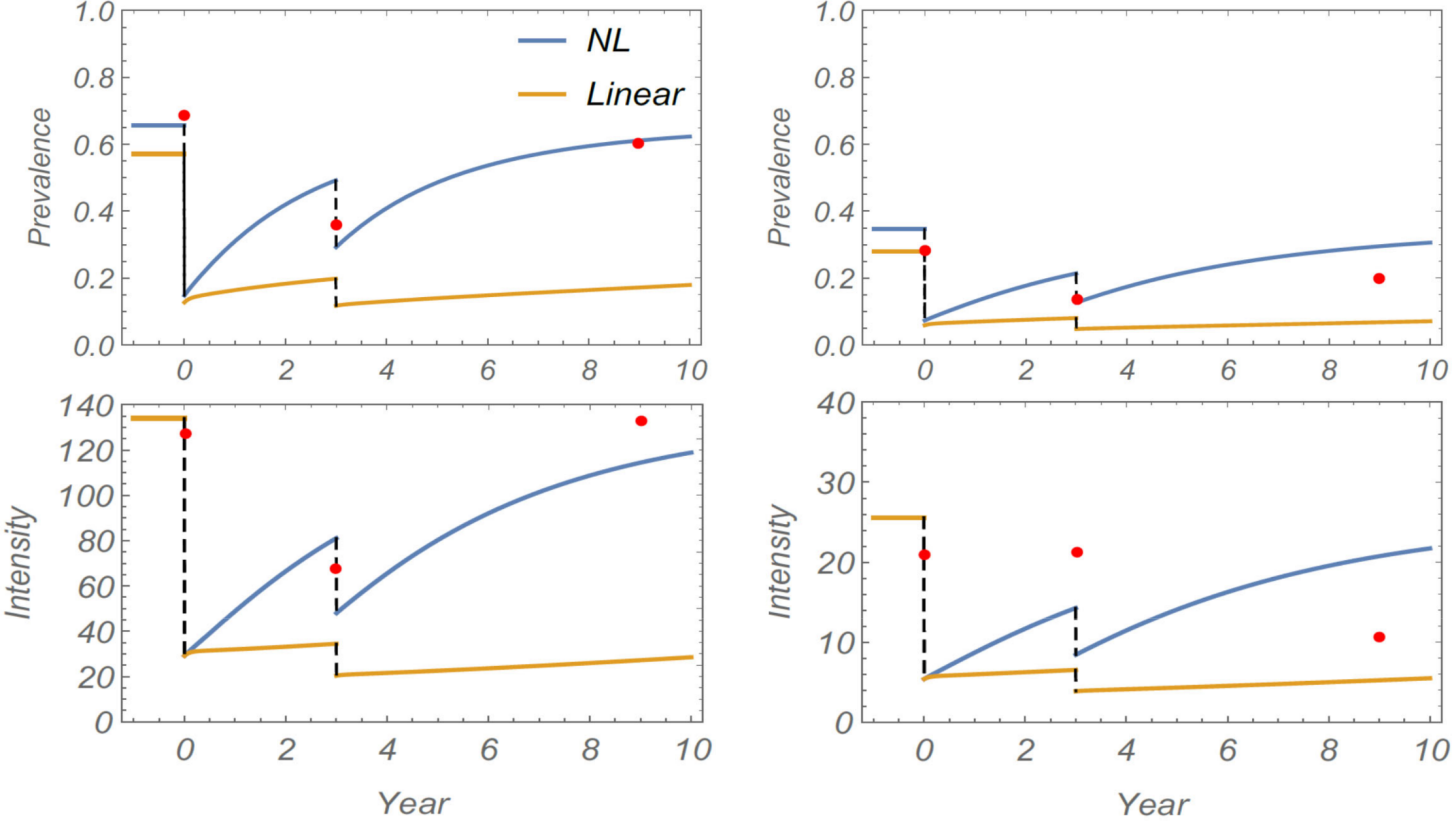
Simulation of 9-year control study for Milalani community using linear M1 (yellow) or non-linear M2 (NL, blue) study model simulations. Model comparison with observed field data (red dots) for *S*. *haematobium* infection prevalence (top graphs) and mean intensity in eggs per 10 mL urine (bottom graphs). The left graphs indicate results for children, the right graphs indicate results for adults. Model parameters of Table 3 were used for both systems.

To assess prediction potential of linear and nonlinear models, both systems were fitted to the baseline infection dataset (2001). As explained in Methods, this yields a posterior ensemble of best-fit calibrated human parameters (*λ*,*k*,*ρ*). We then sampled random choices from this posterior ensemble, along with three additional environmental inputs, (*ω*,*b*,y*), to get the estimated transmission parameters for M1 and M2 (see Table 3). Each virtual community (parameter choice) was simulated over a 9-year period subject to two MDAs. Typical model outcomes are shown in Fig 4, with comparison to observed field data. On both follow-up years (2003, 2009), we observed significant relapse toward pre-control (endemic) levels of infection. Of the two calibrated models, the nonlinear M2 was able to reproduce this pattern for child and adult groups. However, the M1 model did not capture post-treatment prevalence values with its slower intrinsic relapse rate.

**Fig 5:**
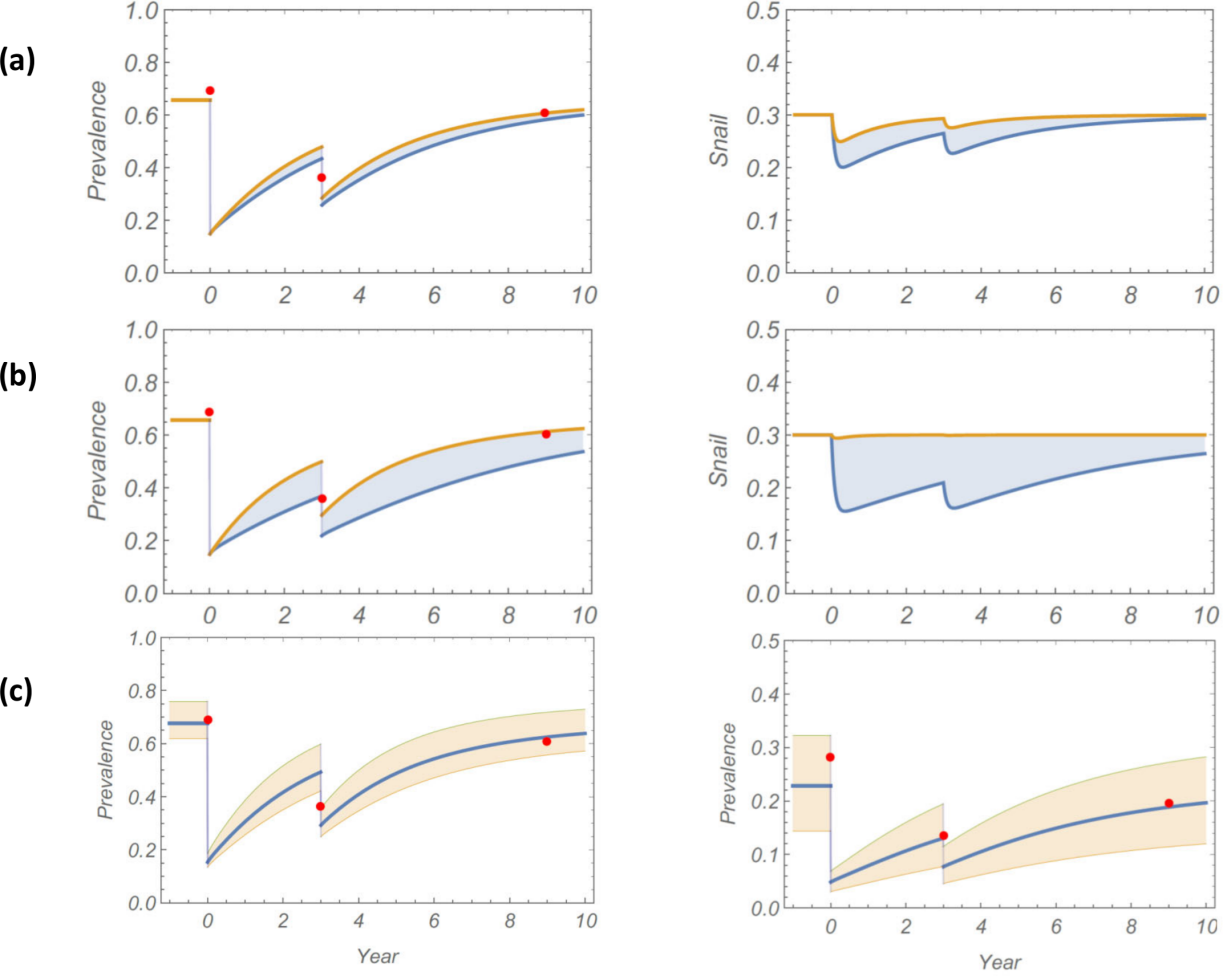
Sensitivity analysis: Solution envelopes and mean history of model M2 for different parameter sampling. Panel (a) the blue shaded regions indicate the full range of predicted outcomes obtained by varying 0.5 < ω < 5, but with fixed *b* = 1, and the (λ, ρ, *k*) inputs of Table 3. Panel (b) indicates the range of outcomes obtained by varying 0.5 < *b* < 4, with fixed ω = 1, and the (λ, ρ, *k*) inputs of Table 3. For Panel (c), pink shading indicates the range of outputs with fixed (ω = 1, *b* = 1) but with random posterior sampling of (λ_*i*_, ρ_*i*_, *k*_*i*_). Panels (a) and (b) show child (left column) and snail (right column) prevalence histories. Panel (c) shows child (left side) and adult (right side) human prevalence values, respectively. In all cases the observed field data points lie within the prediction envelopes.

We again tested parameter sensitivity for robustness of our predictions. This test was run separately for three environmental inputs: i) the relative exposure factor was varied in the range 0.5 < ω < 5, ii) the child transmission rate was varied in the range 0.5 < *b* < 5 (for M2), and iii) a random variation of best-fit panel parameter inputs (*λ*_*i*_,*ρ*_*i*_,*k*_*i*_) of the calibrated community was used in each replicate simulation. In each case, an ensemble of 9-year histories was simulated. Solution envelopes of these ensembles along with their mean path are plotted in Fig 5 (panels *a*, *b*, and *c*). The envelopes are less sensitive to relative exposure factor *ω*, but child transmission *b* had more pronounced effect. The uncertainties due to human inputs {(*λ*_*i*_,*ρ*_*i*_,*k*_*i*_)}, come from the baseline posterior calibration, as shown in panel (c) of Fig 5. In all cases, observed data points lie within prediction envelopes.

### Transition between linear and nonlinear behavior

**Fig 6:**
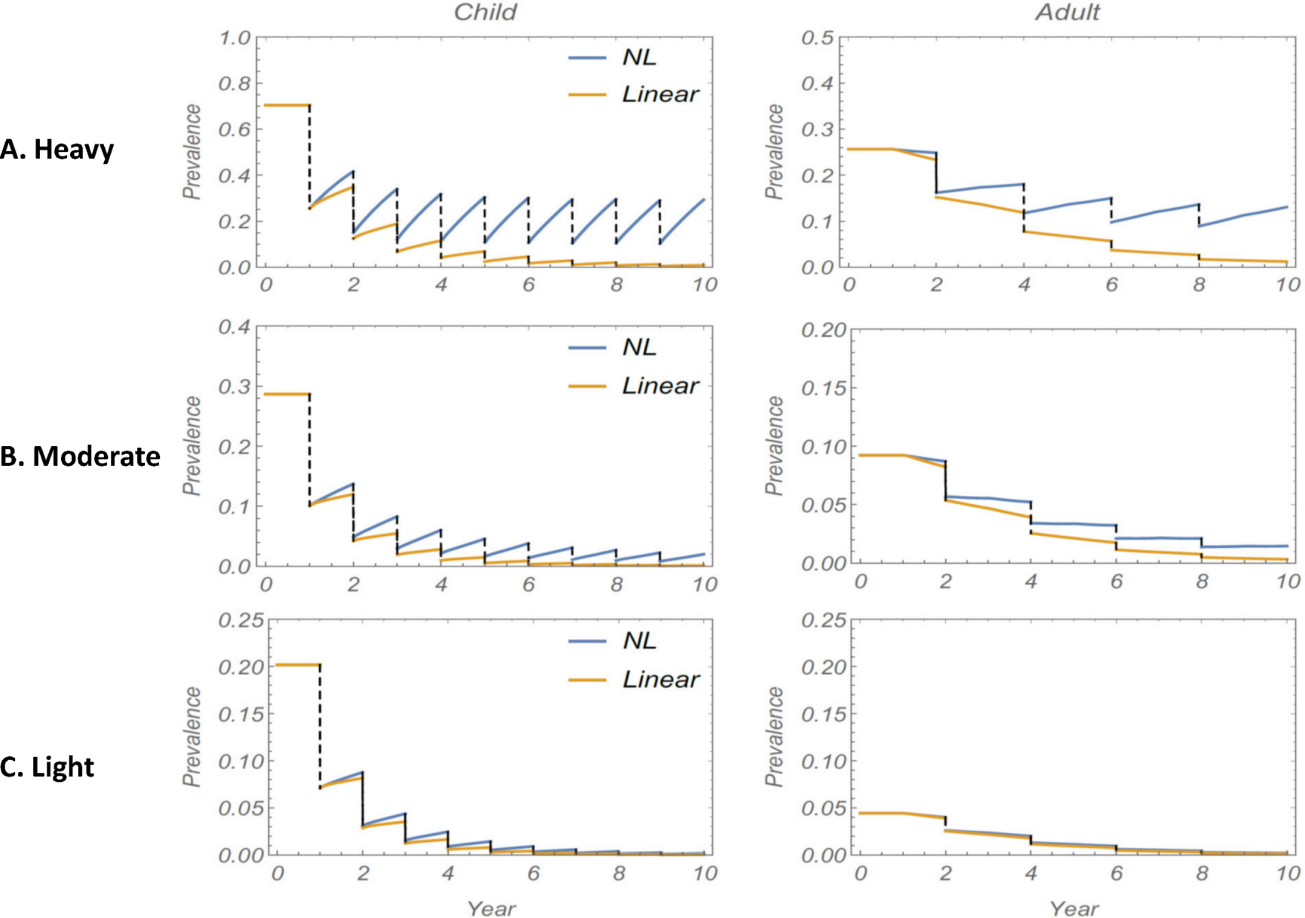
Comparison between M1 and M2 projections for a 10-year MDA regimen in three classes of transmission community. The three plots (A)-(C) show the difference between using a linear (M1) vs. a non-linear (M2) snail FOI input in simulations of MDA program prevalence outcomes in high transmission setting ((A), top panels), moderate transmission settings ((B), middle panels) or low transmission settings ((C), bottom panels) for children (left side graphs) and adults (right side graphs). M1 (linear, yellow lines) and M2 (NL, blue lines) projections diverge significantly in (A), but are very close in in low transmission settings (C), where both projections follow a similar decay pattern.

As discussed earlier in Methods, nonlinear snail FOI becomes approximately linear at low levels of human infectivity. To explore the effect of a reduced transmission environment on long term MDA, we subjected three sample communities with heavy (H), moderate (M) or light (L) transmission intensity, respectively, to the same 10-year control regimen, and compared projected prevalence outcomes for M1 vs. M2 simulations (infection prevalence). Fig 6 shows the comparative results. The difference in simulations is unambiguous for the high risk community, where M1 predicts gradual decline towards elimination, whereas M2 shows strong rebound to moderately high prevalence levels (15–30% for children) each year. For moderate risk areas, the two curves for M1 and M2 are closer, although M2 still predicts a persistent cycle of reinfection. For the low risk community (L) the discrepancy between models appears marginal, with M1 and M2 closely following each other.

## Discussion

In this modeling study, we systematically compared two model structures for *Schistosoma* transmission to better understand the importance of non-linear snail vector dynamics for model prediction of long-term intervention outcomes. We calibrated two transmission models with identical human host inputs but different human-to-snail transmission coupling—a conventional model with linear FOI assumption (M1) and a more complex model assuming a nonlinear saturable FOI for snails (M2)–using longitudinal data collected in coastal Kenya [10, 42]. We subjected both models to a series of numeric experiments simulating different MDA regimens, and found marked differences in long-term epidemiologic predictions. The conventional M1 model predicted efficient control (reaching targeted reductions, then elimination) after relatively few rounds of MDA, even in the face of low or moderate treatment coverage levels. The proposed M2 model, however, found many settings to be highly refractory to MDA treatment impact, with persistent *Schistosoma* re-infection even with high treatment coverage levels. In the model validation, we found the M2 model with its non-linear snail FOI formulation to be more reflective of empirically observed data [10, 29]. Going forward, these findings have clear implications for program monitoring and evaluation and future control implementation for schistosomiasis control, suggesting that a non-linear FOI function should be incorporated for more realistic projections in future *Schistosoma* transmission modeling.

Empirical evidence from other host-pathogen systems [33, 38, 50–53] suggest that there is likely to be a continuum in transmission kinetics that must be considered when modeling the observed transmission patterns found in settings where host numbers and distribution are varied. Although they are more complex and require more data, more nuanced modelling systems are expected to yield better understanding of parasite dynamics and the impact of control interventions [33]. Previous modeling work on *S*. *japonicum* transmission by Liang and colleagues [54] has incorporated multiple human risk groups identified by location and occupation, as well as seasonal aspects of snail reproduction and development. When calibrated against field data, this model more accurately projected the re-emergence of infection in high-risk communities when MDA and other interventions were stopped. Prediction of ‘bounce-back’ risk will be essential in determining the design of follow-up surveillance programs as local elimination is attempted. As noted above, the accurate calibration of such models requires more information about the control areas. However, the greater precision of model projections should improve the efficiency of program interventions [54].

In the presence of nonlinear FOI, a relatively small infective human host pool can exert a disproportionate, leveraged effect on snail infection. Hence, even a steep drop of human infectivity post MDA may result in only a marginal drop of snail infections, and this phenomenon, in turn, may result in a vigorous rebound or human infection to pre-treatment levels as noted in the SCORE project persistent hotspots [28, 29]. In our analysis, we have used independent longitudinal data from communities in rural Kenya to formally compare the proposed non-linear snail FOI models with more conventional models to understand the impact of this effect on long-term model prediction.

In our study system, the concept of a nonlinear, saturable pattern for snail FOI in *Schistosoma* transmission environments (as proposed in the M2 model) has biological plausibility: i) Water contamination occurs in pulses, as infected humans only intermittently contaminate their environment with urine or feces [55]. Human treatment coverage is non-random, with people who are non-adherent to MDA perhaps the most likely ones to contaminate the snail environment (i.e., as effective superspreaders); ii) the miracidia that hatch from contaminating eggs selectively home onto local vector snails in order to infect them [56], iii) because of substantial asexual reproduction of the sporocyst, each infected intermediate host snail has the potential to release thousands of infective cercariae [57, 58], and iv) cercariae sense human skin lipids, and preferentially swim toward any persons coming into contact with affected water bodies [59]. These nonlinear features all bias the transmission process in favor of higher levels of human infection and post-MDA reinfection. Specifically, this means that the extra-human phase of *Schistosoma* transmission is not a random, mass action process, although, for simplicity’s sake, many current models of transmission have assumed that it is.

The coupled human-snail transmission dynamics in a model of schistosomiasis transmission are driven by two FOI: human-to-snail (Λ), and snail-to-human (λ). Each FOI is dependent on its source population size and infectivity, and given the predictive limitations of conventional models, our findings suggest that future models should include an updated accounting of these parasite invasion processes. The two obligate trematode hosts (human and snail) are treated differently in mathematical models of schistosome transmission but their FOIs are often assumed to be linear functions of the combined host infectivity. While such an assumption appears justified for human FOI, λ, snail FOI Λ requires more careful elaboration. In our current analysis, we derived a nonlinear saturable snail FOI function, which embodied several essential environmental (e.g. type of water source, sanitation), demographic (e.g. age distribution), and behavioral inputs (e.g. contact with water, defecation practices), including human/snail population densities (*H*, *N*) and their contact/exposure rates. Given the difficulty of empirically measuring many of these aspects, we calibrated a composite estimate of FOI that reflected many complex and often heterogeneous factors. The conventional linear and proposed nonlinear functions were approximately equal at low levels of human contagion, where the linear FOI could be viewed as an adequate approximation of what is actually a nonlinear Λ. However, the two FOI versions diverged at higher levels of contagion, and so yielded very different transmission parameter estimates when fitted to the same human-snail infection data. The M1 and M2 models, based on the two different systems, also responded differently to strong perturbations, as occurs with MDA interventions; the M2 models predicting substantially faster post-MDA rebound as compared to M1 models. The human part of our present coupled system analysis employed SWB methodology [39, 41–43], but the qualitative conclusions of the M1-M2 comparison would remain true for other transmission models, including MacDonald-type MWB models [24, 45].

Only the nonlinear (M2) was able to accurately reproduce the strong rebound of infection seen in the dataset in years 3 and 9 of the Kenya project. This would predict that such communities will be resilient to any attempts at targeted elimination of transmission. In many cases the temporal differences between the two model systems (M1 & M2) were large, in that M1 community model projections typically achieved control targets over a short time-span with moderate effort, compared to M2 models, where infection was projected to persist much longer and to require extended treatment intervention. In a separate project, we have explored, in greater depth, possible elimination strategies using combined MDA and environmental snail control, and we predict that the only way to achieve target reduction in high transmission communities would be via implementation of additional environmental interventions, *e*.*g*. combining MDA with molluscicide-based snail control [11, 44].

For the nonlinear M2 system, three factors contribute independently to snail FOI estimation, accounting for a variety of MDA responses ranging from near-linear, efficient reduction /elimination in lower prevalence communities, to a highly resilient “locked” pattern of reinfection, whereby each MDA-mediated drop in prevalence is matched by post-treatment rebound. This latter feature could provide a key to the hotspot phenomenon observed in many control programs (see, *e*.*g*. [28, 29]). Indeed, it can explain why adjacent communities with near identical baseline human infection can produce divergent MDA responses based on variations in their local snail environment and in human behavior [60]. Importantly, while the proposed non-linear model demonstrates improved predictive value, this benefit should be balanced with the need for additional community data and more complex parameter estimation. The principle finding of this study is that a relatively simple non-linear function, on average, outperforms a linear function even when considering parameter uncertainty.

Our analysis suggests a defining role of transmission environment (and its resultant snail FOI) for predicting MDA control outcomes. The heterogeneity and connectedness across *Schistosoma* transmission landscapes [16, 45, 61], along with substantial parasite replication in the snail host, appear to make *Schistosoma* infection control much more challenging than for the filarial parasites that are transmitted by insect vectors [3, 4]. In particular, MDA-based ‘transmission control’ for schistosomes will be particularly fragile in the face of persistent non-adherence to treatment (or sanitation) by a small group of infected residents or migrants [44, 45, 62].

In summary, there are substantial complexities in the human and snail factors that can affect *Schistosoma* transmission dynamics and related predictions of MDA-based schistosomiasis control outcomes. This study finds that nonlinear human-snail coupling (FOI) can improve model prediction. Although other model structures could also provide broad agreement with the data, nonlinear snail FOI could provide a plausible explanation of strong MDA resilience (hotspots) observed in the SCORE studies and the observed heterogeneous community responses reported elsewhere [28, 29]. The present work will motivate future studies to apply these ideas to connected human-snail environments (see [14], [63]), and to the analysis of recent control datasets to develop tools to more accurately predict hotspots and explore strategies for their efficient control.

## Supporting information

S1 File Details of the stratified worm burden model and its calibration. (PDF)
