# Supplementary material for: The human-snail transmission environment shapes long term schistosomiasis control outcomes: Implications for improving the accuracy of predictive modeling
Source: PLoS Negl Trop Dis. 2018 May 21;12(5):e0006514. doi: 10.1371/journal.pntd.0006514 (PMC5983867; doi:10.1371/journal.pntd.0006514)
Supplement: S1 File — (PDF) [file pntd.0006514.s001.pdf]

Supporting Information S1 File: The Stratified Worm Burden (SWB) model and its calibration.

A. Human - snail transmission system

The stratified worm burden (SWB) approach to modeling *Schistosoma* transmission stratifies the at-risk human host population by their worm burdens, $H = \sum_{m \geq 0} h_m(t)$, each stratum carrying m worms/person (more generally between $m\Delta w \leq V < (m+1)\Delta w$, with specified worm step $\Delta w > 1$). The transition among strata is described by matrix $M(\lambda, \gamma, \mu)$, and age/group specific source term $\vec{S} = \{S_m(t)\}$ (see [1-3])

$$\frac{d\vec{h}}{dt} = M(\lambda, \gamma, \mu) \cdot \vec{h} + \vec{S} \tag{1}$$

The parameters of matrix *M* include snail-to-human force of infection (FOI), λ, the human population turnover rate, μ, and worm mortality, γ. The infectivity of the SWB within the human population is determined by the mean mated worm count (MMC), ϕ_m, in each stratum, and their weighted mean across strata,

$$\Phi = \sum_{m>0} h_m \phi_m \tag{2}$$

(see, *e.g.* [2, 4-8]). Alternatively, one can simplify and assume a specific worm distribution such as a negative binomial distribution with mean *w* and aggregation constant, *k*. (e.g., the NB(*w,k*) in a MacDonald-type mean worm burden (MWB) model [9]), and get a closed functional form of $\Phi(w,k)$. In either case, MWB or SWB, human infectivity is the product of worm fecundity, ρ, and MMC,

$$E = \rho \Phi \tag{3}$$

In contrast to the MWB NB assumption for worm/person distribution, in our SWB approach, no a priori assumption is made about the worm distribution in the human host community. Egg release by mated females and individual hosts still depends on age-specific worm fecundity, ρ, and MMC ϕ_m (for the h_m - stratum). In our approach, a host in *m*-th stratum is assumed to release random daily egg amounts following a negative binomial (NB)-distribution with mean

$E_m = \rho \phi_m$, and aggregation parameter $k_m = k \phi_m$ [3, 10, 11]. So any test pool of an SWB community with population strata $\{h_m\}$ is viewed as random draw of the NB-mixture distribution with weights h_m .

At the endemic (equilibrium) state, strata variables $\{h_m(\lambda)\}$ are determined by age-specific FOI λ . Hence mean mated count $\Phi(\lambda)$ and human infectivity $E(\lambda)$ become functions of λ . ***Figure 2*** illustrates typical equilibrium distributions for 3 sample villages.

The **snail** population-transmission model employed in our analysis was developed in earlier papers [3, 10, 12]. Here we use a simplified (S-I) version made of susceptible prevalence variables $x + y = 1$, assuming a stationary snail environment,

$$\frac{dy}{dt} = \Lambda(1-y) - \nu y \tag{4}$$

Snail-to-Human FOI, λ - the human per capita rate of worm accumulation, depends on i) the intermediate larval stage (cercaria) density, C , in local water bodies, ii) the rate of human exposure to affected water bodies (contact rate), ω , and iii) human susceptibility to infection, α (probability of worm establishment/ water contact),

$$\lambda = \alpha \, \omega C = A y \tag{5}$$

-a linear function of infected snail prevalence.

Human-to-Snail FOI, Λ , depends on parasite miracidial density, M , snail density, N , snail susceptibility (probability of successful invasion), β , and the maximal rate of snail invasion by miracidia, Λ_0 .In the present model, we propose the following nonlinear (saturated) function for Λ,

$$\Lambda = \Lambda_0 \left(1 - e^{-\beta M/N}\right) \tag{6}$$

Fraction $\left(1 - e^{-\beta M/N}\right)$ is derived from hypothesized Poisson distribution of the “miracidia per snail” variable with mean value M / N . It represents the probability of one or more successful

invasions. Miracidial density, $M = bHE$, depends on human host population size *H*, and human infectivity *E,* so

$$\Lambda(E,N) = \Lambda_0\left(1 - e^{-b\,EH/N}\right) \quad (7)$$

Maximal invasion rate is a function of snail density *N*, and other biological/environmental inputs (see, *e.g*. [13]). However, in the current analysis we treat it as a single (uncertain) parameter to be estimated from other transmission inputs.

The nonlinear function $\Lambda(E,N)$ (7) can be approximated by the linear form, $\Lambda \approx BE$, provided exponent $bEH/N \ll 1$, that is, the combined "human contagion" is small relative to snail density. Such a linear assumption for snail FOI was commonly used in past transmission modeling (*e.g.* [1, 4, 14, 15]). The resulting transmission coefficient B, is then proportional to human population size, *H,* and their water contact rate ω.

Human infectivity, *E,* in equation (7) depends on host population makeup and age –specific exposure/ contamination patterns of different population age groups (children (C) and adults (A)). A single SWB has $E = \rho\Phi(\lambda)$; for mixed population groups C and A, infectivities $\{E_C, E_A\}$ are weighted in proportion to population fractions (H_i), and relative (adult vs. child) exposure factors, $\omega = \omega_A/\omega_C$ [3, 10]. The net result is combined human infectivity

$$E(\omega) = \rho_C\left(H_C\,\Phi(\lambda_C) + \frac{\rho_A}{\rho_C}\omega H_A\,\Phi(\lambda_A)\right). \quad (8)$$

-function of calibrated human parameters $\{(\lambda_i, \rho_i)\}$ drawn from the posterior ensemble of best fit parameters based on our calibration (explained below).

B. Coupled human-snail system and model calibration

Human and snail equations (1) - (4) are coupled via two transmission coefficients *A* (snail-to-human) and B (human-to-snail). The *A* -coefficient is included in the human FOI expression $\lambda = Ay$ (infected snail prevalence), and coefficient B is included in the exponent of the nonlinear snail FOI, Λ. The details of model calibration and estimated parameter values are explained in previously published papers [3, 10]. It proceeds in two steps: (i) *human calibration for* (λ_i, k_i, ρ_i) which are snail-to-human FOI, aggregation, and worm fecundity, estimated from

test data (stool eggs per gram (epg, *S. mansoni*) or eggs per 10 mL by urine filtration (*S. haematobium)*); (ii) *transmission calibration*, for other coefficients (see Table A1).

Model inputs include

(i) **Human parameters**: age-specific triplets (λ_i, k_i, ρ_i), $i = \{C, A\}$ which enter human infectivity function, $E_i = \rho_i \, \Phi(\lambda_i)$. Table A2 gives the results of human calibration (parameters statistics) for 3 sample communities, and **Figure *1*** shows their marginal distributions

(ii) **Snail inputs**: infected prevalence y^*

(iii) **Environmental/behavioral inputs**: *b* – child transmission coefficient, ω - relative (adult/child) exposure contamination

Specifically, for A, coefficients are estimated from calibrated human FOI λ_i and (known or hypothesized) infected snail density *y*

$$A_i = \frac{\lambda_i}{y} \; ; \; i = \{C, A\} \tag{9}$$

For linear form of snail FOI (used in model M1), transmission coefficient *B* is estimated from the equilibrium snail equation (4) as

$$B(\omega) = \frac{\nu \, y^*}{(1 - y^*) E(\omega)} \tag{10}$$

with combined infectivity function **Error! Reference source not found.**.

For the nonlinear form of snail FOI (used in model M2) calibration proceeds differently: instead of estimating the pairs (A_i, B), we estimate (A_i, Λ_0),

$$\Lambda_0(b, \omega) = \frac{\nu \, y^*}{(1 - y^*)\left(1 - e^{-b\,E(\omega)}\right)} \tag{11}$$

We sample model uncertainties to provide estimates of variance, and generate a 95% uncertainty interval (UI) in the transmission projection for control interventions.

Table A1: Summary of model parameters

<table>
<tr><td></td><td>Known, fixed</td><td>Calibrated</td></tr>
<tr><td>Human</td><td>Demographic inputs
<table><tr><td></td><td>C</td><td>A</td></tr><tr><td>Population fractions</td><td>0.49</td><td>0.51</td></tr><tr><td>Host turnover/year</td><td>0.06</td><td>0.025</td></tr><tr><td>Worm mortality/year</td><td>0.2</td><td>0.25</td></tr></table></td><td>Posterior distributions of $\{\lambda^i, \rho^i, k^i\}$, for age-groups $i = \{C, A\}$, estimated from community test data ([16, 17])</td></tr>
<tr><td>Snail</td><td>Mortality: $\nu = 4$ /year,
Infected prevalence (baseline endemic value)[a]: $y^* = .3$ (range $.15 - .45$)</td><td>Estimated
Max invasion (FOI) rate: Λ_0</td></tr>
<tr><td>Environment/ behavior</td><td>Basic (child) transmission rate: $.5 < b < 5$
Relative exposure/contamination rates (“adult/child): $.5 < \omega < 1.5$</td><td>Transmission coefficients $A_i = \lambda^i / Z$</td></tr>
</table>

[a] These ranges are broadly consistent with published data [16, 18]

Table A2: Calibrated posterior parameters for 3 Kenyan communities (H – heavy, M – moderate, L –light)

Community		λC	λA	kC	kA	ρC	ρA
H	Min	1.8	0.51	0.018	0.014	9.8	4.6
	Max	5.9	1.7	0.083	0.045	35.	12.
	Median	2.8	0.85	0.044	0.026	20.	8.3
	Mean	3.1	0.91	0.046	0.027	21.	8.4
	SD/Mean	0.31	0.31	0.36	0.3	0.3	0.23
M	Min	0.54	0.18	0.049	0.007	40.	2.9
	Max	0.69	0.95	0.077	0.048	55.	14.
	Median	0.6	0.33	0.063	0.023	47.	8.2
	Mean	0.61	0.37	0.063	0.024	47.	8.2
	SD/Mean	0.059	0.42	0.11	0.42	0.079	0.33
L	Min	0.37	0.035	0.045	0.016	28.	3.3
	Max	0.51	0.2	0.084	0.12	44.	14.
	Median	0.43	0.067	0.064	0.059	36.	8.
	Mean	0.43	0.077	0.064	0.062	36.	8.2
	SD/Mean	0.078	0.44	0.14	0.43	0.11	0.32

Captions

Figure 1: Marginal distributions of calibrated posterior distribution for 3 sample communities from coastal Kenya [20]. Demographic makeup consists of children (0-20) adult (20+). For each group, EPG test results were fitted to its (λ, ρ, k)- parameters

Figure 2: Typical endemic (baseline) SWB distributions in 3 sample communities based of calibrated posterior values $\{\lambda_C, \lambda_A\}$

Figures

Figure 1.

H

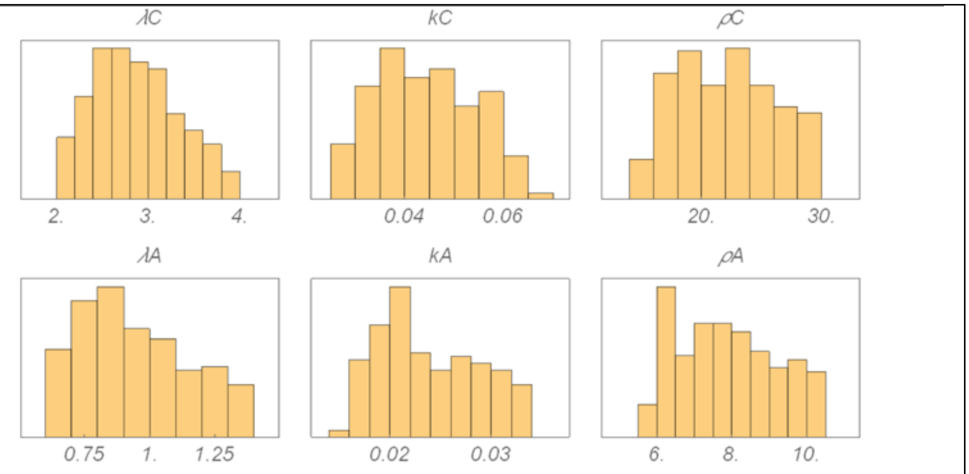

M

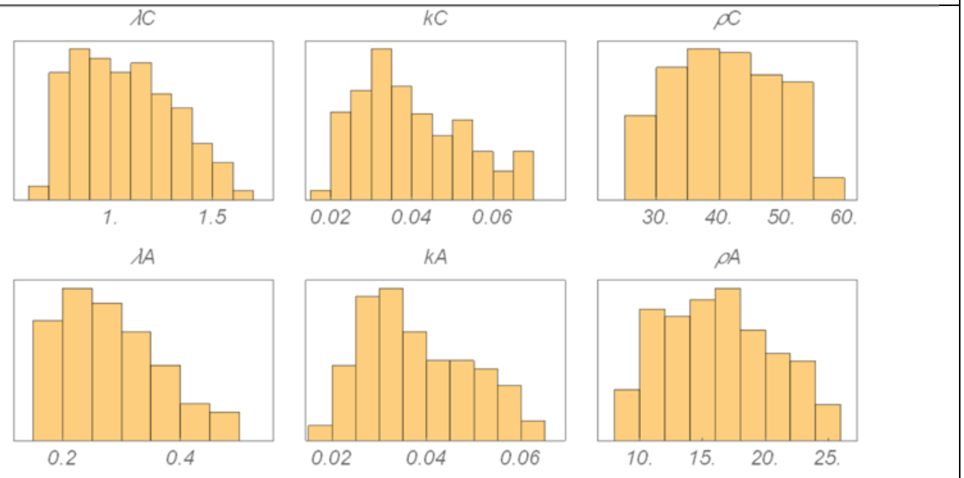

L

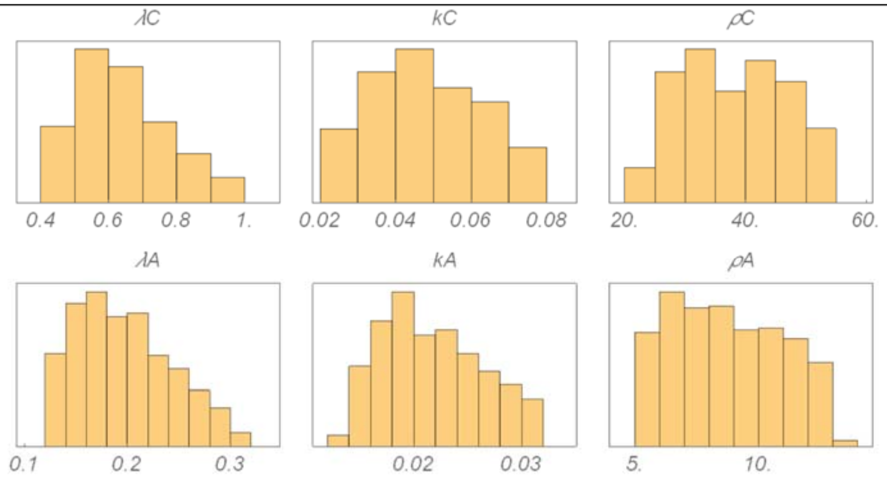

Figure 2.

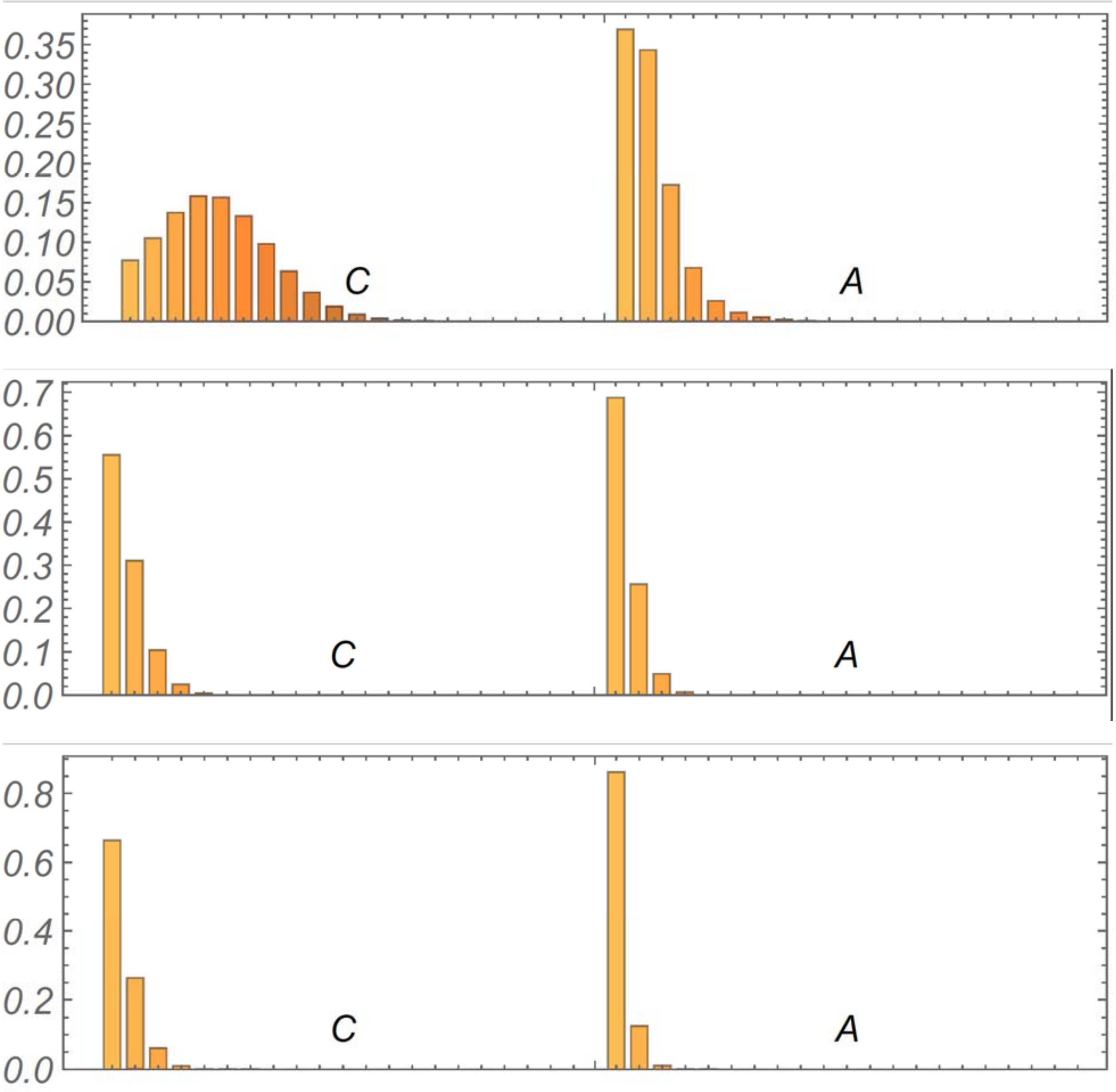
0.35
0.30
0.25
0.20
0.15
0.10
0.05
0.00
C
A
0.7
0.6
0.5
0.4
0.3
0.2
0.1
0.0
C
A
0.8
0.6
0.4
0.2
0.0
C
A

References

1. Gurarie D, King CH, Wang X. A new approach to modelling schistosomiasis transmission based on stratified worm burden. Parasitology. 2010;137(13):1951-1965. Epub 2010/07/14. doi: 10.1017/S0031182010000867.
2. Gurarie D, King CH. Population biology of *Schistosoma* mating, aggregation, and transmission breakpoints: More reliable model analysis for the end-game in communities at risk. PLoS One. 2014;9(12):e115875. doi: doi: 10.1371/journal.pone.0115875.
3. Gurarie D, King CH, Yoon N, Li E. Refined stratified-worm-burden models that incorporate specific biological features of human and snail hosts provide better estimates of Schistosoma diagnosis, transmission, and control. Parasit Vectors. 2016;9(1):428. doi: 10.1186/s13071-016-1681-4.
4. May RM. Togetherness among schistosomes: Its effects on the dynamics of infection. Mathematical Biosciences. 1977;35:301-343.
5. May RM, Woolhouse ME. Biased sex ratios and parasite mating probabilities. Parasitology. 1993;107(Pt 3):287-295.
6. Nasell I. Mating for schistosomes. J Math Biol. 1978;6(1):21-35.
7. Nåsell I, Hirsch WM. The transmission dynamics of schistosomiasis. Communications on Pure and Applied Mathematics. 1973;26(4):395-453.
8. Shaw DJ, Grenfell BT, Dobson AP. Patterns of macroparasite aggregation in wildlife host populations. Parasitology. 1998;117 (Pt 6):597-610.
9. MacDonald G. The dynamics of helminth infections, with special reference to schistosomes. Trans R Soc Trop Med Hyg. 1965;59(5):489-506.
10. Gurarie D, Yoon N, Li E, Ndeffo-Mbah M, Durham D, Phillips AE, et al. Modelling control of *Schistosoma haematobium* infection: predictions of the long-term impact of mass drug administration in Africa. Parasit Vectors. 2015;8(1):529. doi: 10.1186/s13071-015-1144-3.
11. Gryseels B, De Vlas SJ. Worm burdens in schistosome infections. Parasitol Today. 1996;12(3):115-119. Epub 1996/03/01. doi: 0169475896806715 [pii].
12. Gurarie D, King C, Yoon N, Alsallaq R, Wang X. Seasonal Dynamics of Snail Populations in Coastal Kenya: Model Calibration and Snail Control. Advances in Water Resources. 2016.
13. Gurarie E, Ovaskainen O. Towards a general formalization of encounter rates in ecology. Theoretical Ecology. 2013;6(2):189-202.
14. Anderson RM, May RM. Infectious Diseases of Humans. Dynamics and Control. New York: Oxford University Press; 1991. 467-496, 507-420 p.
15. Wang S, Spear RC. Exploring the Contribution of Host Susceptibility to Epidemiological Patterns of Schistosoma japonicum Infection Using an Individual-Based Model. The American Journal of Tropical Medicine and Hygiene. 2015. doi: 10.4269/ajtmh.14-0691.
16. Muchiri EM, Ouma JH, King CH. Dynamics and control of *Schistosoma haematobium* transmission in Kenya: an overview of the Msambweni Project. Am J Trop Med Hyg. 1996;55(5 Suppl):127-134.
17. Bisanzio D, Mutuku F, Bustinduy AL, Mungai PL, Muchiri EM, King CH, et al. Cross-sectional study of the burden of vector-borne and soil-transmitted polyparasitism in rural communities of Coast Province, Kenya. PLoS Negl Trop Dis. 2014;8(7):e2992. doi: 10.1371/journal.pntd.0002992.

18. Sturrock RF. Field studies on the transmission of *Schistosoma mansoni* and on the bionomics of its intermediate host, *Biomphalaria glabrata*, on St. Lucia, West Indies. Int J Parasitol. 1973;3(2):175-194.
19. Webbe G. Quantitative studies of intermediate host populations in the transmission of schistosomes. Proc R Soc Med. 1968;61(5):455.
20. Kariuki HC, Clennon JA, Brady MS, Kitron U, Sturrock RF, Ouma JH, et al. Distribution patterns and cercarial shedding of *Bulinus nasutus* and other snails in the Msambweni area, Coast Province, Kenya. Am J Trop Med Hyg. 2004;70(4):449-456.
